# Fixation Duration and Pupil Size as Diagnostic Tools in Parkinson’s Disease

**DOI:** 10.3233/JPD-202427

**Published:** 2021-04-13

**Authors:** Panagiota Tsitsi, Mattias Nilsson Benfatto, Gustaf Öqvist Seimyr, Olof Larsson, Per Svenningsson, Ioanna Markaki

**Affiliations:** a Department of Clinical Neuroscience, Neuro, Karolinska Institutet, Stockholm, Sweden; b Center of Neurology, Academic Specialist Center, Region Stockholm, Sweden; c Department of Clinical Neuroscience, Eye and Vision, Karolinska Institutet, Stockholm, Sweden; d Neurology Department, Karolinska University Hospital, Stockholm, Sweden

**Keywords:** Parkinson’s disease, eye-tracking, fixation, pupil size, eye movements

## Abstract

**Background:**

Visual and oculomotor problems are very common in Parkinson’s disease (PD) and by using eye-tracking such problems could be characterized in more detail. However, eye-tracking is not part of the routine clinical investigation of parkinsonism.

**Objective:**

To evaluate gaze stability and pupil size in stable light conditions, as well as eye movements during sustained fixation in a population of PD patients and healthy controls (HC).

**Methods:**

In total, 50 PD patients (66% males) with unilateral to mild-to-moderate disease (Hoehn & Yahr 1–3, Schwab and England 70–90%) and 43 HC (37% males) were included in the study. Eye movements were recorded with Tobii Pro Spectrum, a screen-based eye tracker with a sampling rate of 1200 Hz. Logistic regression analysis was applied to investigate the strength of association of eye-movement measures with diagnosis.

**Results:**

Median pupil size (OR 0.811; 95% CI 0.666–0.987; *p* = 0.037) and longest fixation period (OR 0.798; 95% CI 0.691-0.921; *p* = 0.002), were the eye-movement parameters that were independently associated with diagnosis, after adjustment for sex (OR 4.35; 95% CI 1.516–12.483; *p* = 0.006) and visuospatial/executive score in Montreal Cognitive Assessment (OR 0.422; 95% CI 0.233–0.764; *p* = 0.004). The area under the ROC curve was determined to 0.817; 95% (CI) 0.732–0.901.

**Conclusion:**

Eye-tracking based measurements of gaze fixation and pupil reaction may be useful biomarkers of PD diagnosis. However, larger studies of eye-tracking parameters integrated into the screening of patients with suspected PD are necessary, to further investigate and confirm their diagnostic value.

## INTRODUCTION

Visual and oculomotor problems in Parkinson’s disease (PD) are among the most common non-motor symptoms reported by patients. Diplopia (double vision), alterations in visuoperception, impairment in contrast, and color vision, as well as visual hallucinations, are some of the usual complaints [1]. Common findings during clinical evaluation are hypometric saccades, hyperreflexivity, increased latency of voluntary saccades, and saccadic intrusions during smooth pursuit eye movements [2]. These symptoms are not specific for PD although the majority appear early in the course of the disease. So far, the use of eye-tracking to assess eye movements has not been part of the routine clinical workup in parkinsonian disorders mainly because, to date, most high-end eye-tracking systems suitable for clinical research have been rather complicated to use by non-experts, making them impractical as an everyday assessment tool in typical clinical settings.

Research on eye movements and oculomotor control in PD has traditionally focused on saccades and saccadic performance using test paradigms like the pro- and anti-saccade task. Small eye movements made during attempted fixation have received less attention although this has started to change in recent years due to the renewed interest in fixational eye movements and their relation to visual perception, attention, and cognition. The role of fixation is to maintain the image of the object of interest in the fovea which is the area of the retina where the visual acuity is best. To keep the target near the fovea, multiple brain structures are recruited. Apart from excitatory activity that stabilizes the eyes, inhibitory processes during fixation prevent saccadic eye movements that would break it. Both cortical (frontal and parietal areas) and subcortical domains (such as the nucleus raphe interpositus and superior colliculus) contribute to this equilibrium [3]. Nevertheless, if the eye was completely stable during the visual fixation of a target, censor adaptation on the retina would lead to blurring and eventually fading of the image. Therefore, corrective microsaccades, tremor, and conjugational drifts are necessary, not only to improve the function of fixation but also to correct errors [4]. Apart from these mini regulatory movements, saccadic intrusions may interrupt fixation. In the case of PD, saccadic intrusions are common, and they appear in the form of square wave jerks (SWJ) that move the eye away from the target during sustained fixation or smooth pursuit. A return saccade back to the target follows the SWJ after approximately 200 ms. The role of the superior colliculus and the fastigial oculomotor region of the cerebellum has been discussed in the genesis of SWJ [5].

Another interesting factor, apart from gaze stability, that can be studied during sustained fixation, while a person is required to maintain focus on a stable target, is the size of the pupil that is affected both by light and cognitive processes that include target detection and attention [6]. Changes in pupil reactivity have already been described in PD and the role of the parasympathetic autonomic nervous system in impaired pupil reflex has previously been discussed [7]. More specifically, it has been shown that cholinergic deficits in PD patients with cognitive impairment may affect the pupil light reflex [8]. Studies on pupil’s reaction to light have yielded rather controversial results due to the role of psychiatric features as well as cognitive impairment but despite the extended literature on light reflex in PD, studies on pupil size in stable light conditions during fixation are scarce.

The purpose of this study was to evaluate gaze stability and pupil size in stable light conditions during sustained fixation in a population of PD patients and healthy controls (HC). Eye-tracking is an objective, non-invasive, and cost-effective analysis method that enables accurate and detailed gaze and eye-movement examination, where processing with advanced algorithms allows for accurate calculation of eye position and movement parameters. It is easy to use in different environments and does not require any verbal, written, or other active actions by the test person.

## MATERIALS AND METHODS

### Participants

Fluent Swedish-speaking PD patients with Hoehn and Yahr (H&Y)≤3 and age-matched healthy volunteers were included in the study. The diagnosis was based on the United Kingdom Brain Bank Criteria [9]. Individuals suffering from eye conditions such as macular degeneration or non-operable/non-corrected cataract were excluded and vision was normal or corrected to normal (visual acuity of a logMAR score≤0.00), regarding both, refraction, and presbyopia. The study was approved by the Stockholm Ethical Committee (DNR: 2018/437-31/2) and participants provided written and oral informed consent, according to the declaration of Helsinki. In total, 50 PD patients (66% males) with unilateral to mild-to-moderate disease (H&Y 1–3, Schwab and England 70–90%) and 43 HC (37% males) were recruited at the Center of Neurology, Academic Specialist Center in Stockholm, Sweden. The participants were clinically assessed with the Unified Parkinson’s Disease Rating Scale (UPDRS) [10] as well as the Montreal Clinical Assessment (MoCA), Mini-Mental State Examination (MMSE), and Frontal Assessment Battery (FAB).

### Apparatus and stimuli presentation

Eye movements were recorded with Tobii Pro Spectrum, a screen-based eye tracker with a sampling rate of 1200 Hz. Data were recorded binocularly, i.e., from left and right eye simultaneously. Stimuli were presented on the native 23.8” Tobii Pro Spectrum screen (EIZO FlexScan EV2451) with pixel resolution 1920×1080 (52.8×29.7 cm). The screen was located approximately 65 cm in front of the participant who was sitting on a steady and comfortable chair in a dimly lit room. Visual acuity was tested with the Landolt C Chart and participants were included only if they received a logMAR score of 0.00 or less, after correcting for refraction and/or presbyopia. The participant was given clear and simple instructions by the examiner to keep his or her eyes focused on the black “dot” (6 mm diameter fixation target subtending approximately 0.5 degrees of visual angle) in the center of a bright white screen. The fixation target was on display for 15 seconds in each trial, after which it briefly disappeared for 5 seconds. Each participant performed eight trials with a total duration of 3 minutes, providing 2 minutes of fixation data for analysis. A 5-point calibration followed by four points of validation was performed on each participant prior to the first trial. Recalibrations were made if deemed necessary by the examiner.

### Data processing

The recorded data were filtered through the Tobii I-VT (Identification by Velocity Threshold) algorithm available in the analysis software Tobii Pro Lab. The primary purpose of the filtering algorithm is to identify fixations and saccades in the raw gaze data. Given a sampling frequency of 1200 Hz, the eye tracker produces a gaze sample approximately every 0.83 ms. The I-VT filter uses a velocity criterion to calculate whether a sequence of raw gaze samples belongs to the same fixation or whether they are part of a saccade in progress.

Since eye-movement patterns may vary substantially between different types of tasks and stimuli, it is often necessary to adapt the fixation filter’s parameter settings to the type and quality of data at hand. We used the algorithm’s default settings in the filtering, with two exceptions: (1) a 5-point running median was used to reduce the level of noise (default: 3-point running median); (2) gap fill-in interpolation was used to fill in short periods of data loss (default: gap fill-in interpolation is inactive). We used the default value for the velocity threshold, which is 30°/s, to allow for relatively short and fast movements to be detected as saccades. It is worth noting that all recordings were subjected to the same filtering, which is standard practice, i.e., the parameters of the I-VT filter were not adjusted to fit each participant or recording individually. After data filtering, a set of eye-movement parameters were computed over the fixation target stimulus for each trial. In line with Castet and Crossland [11], the first 1.5 seconds of each trial were removed before the calculation in order to discard data when the participant was first locating the fixation target in a trial. All computed gaze parameters were based on left and right eye averages. However, when only one eye was found for a data sample, that eye was used in the computation.

Two categories of parameters were computed: sample- and event-based. Sample-based parameters were based on the stream of eye-tracker samples rather than on fixation and saccade events detected by the I-VT fixation algorithm. These measures provide information on the horizontal and vertical gaze position (degrees of visual angle), and bivariate contour ellipse area (BCEA) (square degrees of visual angle). The BCEA measures dispersion as the area of an ellipse encompassing a given proportion P of gaze points. Here we used *P* = 0.682, thus giving the area of the ellipse over which gaze positions were found 68.2% of the time (i.e., dispersion of the gaze about its mean position with±1 standard deviation). Lower BCEA-values indicate higher/better fixation stability. Pupil size (mm) was also computed from the stream of eye-tracker samples. By contrast, event-based parameters, such as fixation duration (sec) and saccade rate (number of detected saccades per second), during fixation, were computed over the detected events, either fixations or saccades and they depend on the I-VT algorithm’s ability to identify them. The mean and median values over the trials were then computed for each participant. Since the median is generally more tolerant to outliers than the mean, eye-movement measures that were aggregated over the trials are given as the medians across the trials. For example, the “mean pupil size” parameter reflects the mean of medians of diameters of the pupil measured during the fixation task.

The gaze parameters primarily attempt to quantify the fixational eye stability during the task. A possible hypothesis is that PD participants are less stable in their fixation than the control group and less able to keep their eyes fixated over time. If true, we would expect, for example, higher dispersion around the mean fixation position, as well as shorter periods of fixation uninterrupted by saccades in the PD group compared to the control group.

### Statistical analysis

Statistical analysis was done with IBM SPSS 25 Statistic Data Editor. Non-parametric tests were used, and the significance level was defined at 0.05. Logis-tic regression analysis was applied to investigate the strength of association of the eye-movement measures, alone or in combination, as predictors of dia-gnosis (HC vs PD), in separate multivariate models, including also sex, age, and cognitive scores. Re-ceiver Operating Characteristic (ROC) curve was plotted to visualize the final model’s separation potential between diagnoses. Spearman correlation was used to investigate the correlation between eye-movement measures and cognitive scores.

## RESULTS

### Participants’ characteristics

In total, 50 PD patients and 43 HC were included in the analysis. Sex distribution differed with more men included in the PD than the HC group (*p* = 0.006), whereas age and years of education did not differ between groups (Table 1). Median disease duration in the PD group was 2 years, corresponding to early-stage disease, as indicated also by the median LEDD (545) and UPDRS part 3 (21 points).

**Table 1:** Clinical and demographical characteristics of PD patients and HC

	HC, N = 43	PD, N = 50	*p*
Sex, male/female	16/27	33/17	**0.006**
Age	63 (16)	64 (10.5)	0.728
Education, years	15 (5)	16 (4)	0.895
Age at onset	61 (12)	NA
Age at diagnosis		62 (11.5)	NA
Years since onset		4 (4.25)	NA
Years since diagnosis		2 (2.5)	NA
LEDD		545 (523.75)	NA
UPDRS part 1		1 (2)	NA
UPDRS part 2		10 (6)	NA
UPDRS part 3		21 (15.5)	NA
UPDRS part 4		2 (3.25)	NA
UPDRS total		36.5 (21.75)	NA
Schwab & England		90 (10)	NA
Comorbidities and treatments, % (n)
Diabetes	2.3 (1)	0	0.5
Atrial fibrilation	2.3 (1)	8 (4)	0.4
Hypertension	23.3 (10)	30 (15)	0.5
Depression	2.3 (1)	14 (7)	0.07
Glaucoma	2.3 (1)	6 (3)	0.6
Prostate hyperplasia/urinary incontinence	2.3 (1)	18 (9)	**0.018**
B12 supplementation	7 (3)	32 (16)	**0.04**
Use of Benzodiazepines	0	8 (4)	0.12
Use of Anticholinergics	0	4 (2)	0.5

Values are reported as medians and interquartile ranges (IQR), apart from the gender ratio that is reported in absolute values. LEDD, Levodopa Equivalent Daily Dose; UPDRS, Unified Parkinson’s Disease Rating Scale; NA, non-applicable.

With regard to cognition, MoCA, MMSE, and FAB scores did not differ significantly between PD patients and HC. However, PD patients had lower score than HC in the visuospatial/executive domain in MoCA (median 4 vs 5; *p* = 0.005; Table 2).

**Table 2:** Cognitive scores of PD patients and HC

	HC,	PD,	*p*
	N = 43	N = 50
MoCA - Visuospatial/executive	5 (1)	4 (2)	**0.005**
MoCA - Naming	3 (0)	3 (0)	0.2
MoCA - Attention	6 (0)	6 (1)	0.5
MoCA - Language	3 (1)	3 (1)	0.3
MoCA - Abstraction	2 (0)	2 (0)	0.4
MoCA - Delayed recall	3 (2)	3 (2)	0.9
MoCA - Orientation	6 (0)	6 (0)	0.2
MoCA Total score	27 (3)	27 (3)	0.2
MMSE Total score	29 (2)	28 (2)	0.4
FAB Total score	18 (2)	17 (3)	0.1

Values are reported as medians and interquartile ranges (IQR). FAB, Frontal Assessment Battery; MMSE, Mini-Mental State Examination; MoCA, Montreal Cognitive Assessment.

Overall, regarding comorbidities and concomitant treatments (Table 1), we only found statistically significant differences regarding prostate hyperplasia and/or urinary incontinence (1 HC vs 9 PD, *p* = 0.018), and B12 supplementation that was more common in the PD group (3 HC vs 16 PD, *p* = 0.04). Only one HC had a diagnosis of diabetes mellitus. In the PD group, four patients where receiving benzodiazepines (three patients received low dose clonazepam for the treatment of REM sleep behavior disorder and one was treated with oxazepam as required against anxiety). Additionally, three patients were treated with anticholinergic medication; two were treated with solifenacine for urinary incontinence and the other one under trihexyphenidyl for the treatment of tremor. Depression was more common in the PD group (1 HC vs 7 PD participants). One HC was treated for glaucoma versus three participants in the PD group.

### Fixation task results

Tables 3 and 4 summarize the fixation task results in PD patients and HC. Regarding the sample-based parameters, comparison of horizontal and vertical gaze position showed significant differences in the median absolute deviation (MAD) and the quartile deviation (QD) of the horizontal gaze position (*p* = 0.023 and *p* = 0.028, respectively; Table 3). It is worth to notice that both standard deviation (SD) and BCEA use the mean in their formula, while MAD and QD are non-parametric estimates, and this could possibly explain the non-significant results in the SD and BCEA comparisons. Also, the horizontal component seems primarily affected, as there are no significant differences in the vertical components of the sample-based gaze measures. Moreover, both mean and median pupil diameter were larger in the HC participants than in PD (*p* = 0.002 and 0.003, respectively).

**Table 3:** Sample-based eye-movement parameters in PD patients and HC

	HC, N = 43	PD, N = 50	*p*
SD gaze point horizontal	0.2 (0.37)	0.32 (0.59)	0.1
SD gaze point vertical	0.34 (0.42)	0.33 (0.57)	0.2
MAD gaze point horizontal	0.09 (0.05)	0.12 (0.2)	**0.023**
MAD gaze point vertical	0.08 (0.06)	0.11 (0.12)	0.1
QD gaze point horizontal	0.1 (0.05)	0.13 (0.27)	**0.028**
QD gaze point vertical	0.09 (0.07)	0.11 (0.19)	0.1
BCEA	0.5 (1.73)	0.8 (3.02)	0.2
Mean pupil size	2.5 (0.3)	2.36 (0.31)	**0.002**
Median pupil size	2.5(0.29)	2.36 (0.31)	**0.003**

Values are reported as medians and interquartile ranges (IQR). BCEA, Bivariate contour ellipse area (square degrees of visual angle); MAD gaze point, Median absolute deviation of the horizontal/vertical gaze position (degrees of visual angle); QD gaze point, Quartile deviation of the horizontal/gaze position (degrees of visual angle); SD gaze point, Standard deviation of the horizontal/vertical gaze position (degrees of visual angle). Mean and median pupil size computed in mm.

Table 4 displays comparisons between event-based parameters computed between the PD and HC populations. Our results showed that during fixation, mean (*p* = 0.007) and median fixation duration (*p* = 0.016), and longest fixation period (*p* = 0.008) were significantly longer in the HC group than the PD group. In accordance with the above, the number of detected saccades during the task was higher in the PD group (*p* = 0.015).

**Table 4:** Event-based eye-movement parameters in PD patients and HC

	HC, N = 43	PD, N = 50	*p*
Mean fixation duration	3.02 (4.25)	1.3 (3.93)	**0.007**
Median fixation duration	2.55 (4.48)	0.73 (5.09)	**0.016**
Longest fixation period	6.1 (5.46)	4.35 (5.34)	**0.008**
Saccade rate	0.44 (0.97)	1.11 (2.49)	**0.015**

Values are reported as medians and interquartile ranges (IQR). Longest fixation period: The maximum period of fixation uninterrupted by saccades, blinks or noise (sec); Mean and median fixation duration computed in sec; Saccade rate: The number of detected saccades per second.

**Fig. 1:**
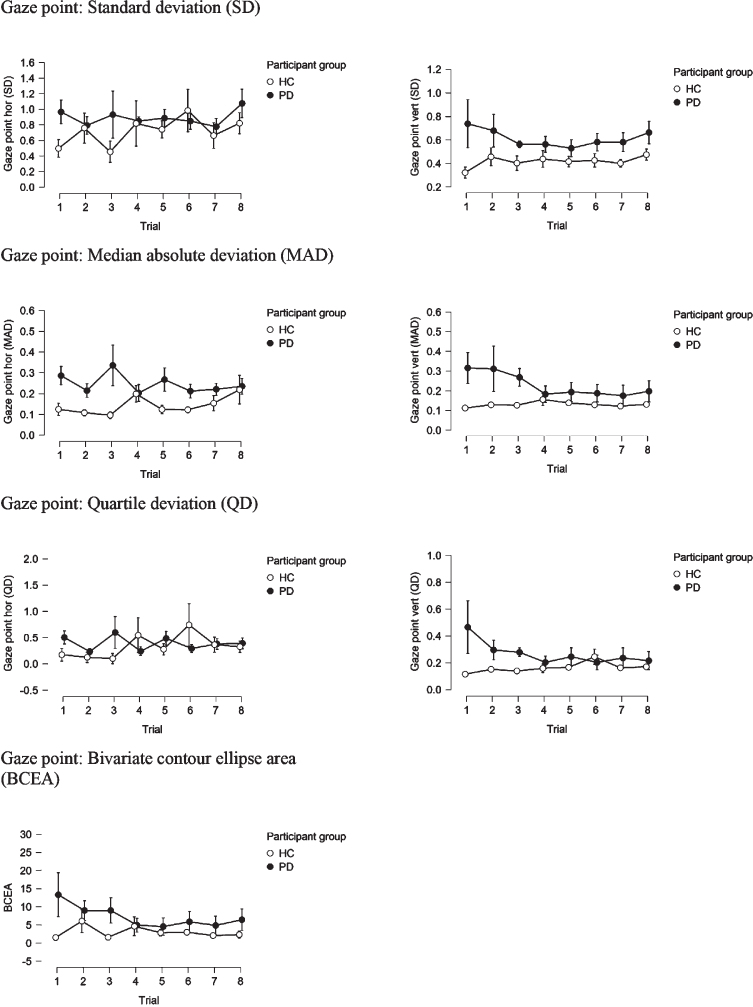
Gaze point parameters on a trial-by-trial basis in PD and HC groups.

**Fig. 2:**
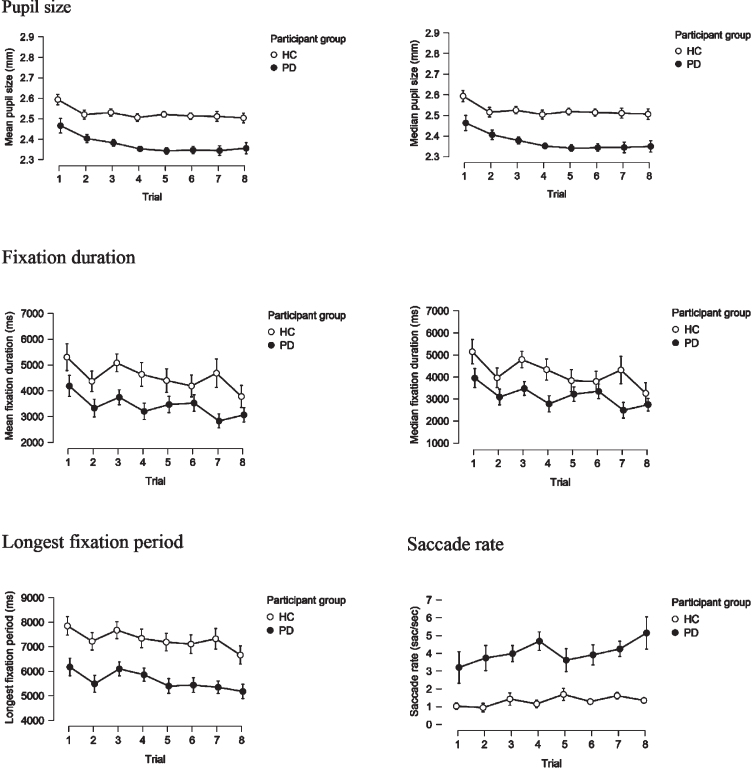
Mean and median pupil size, mean and median fixation duration, longest fixation period and saccade rate, on a trial-by-trial basis in PD and HC groups.

In order to address the fatigue effect, we examined each eye-movement parameter on a trial-by-trial basis for the two groups and compared them schematically. Parameters shown in Fig. 1 are measures of gaze variability/instability, which would be expected to increase in variability, in case fatigue-effect was present, however, this is not observed. Fixation parameters (Fig. 2) would be expected to show a decreasing trend with shorter fixations over time as participants become increasingly tired and find it harder to maintain fixation. This is observed to some extent, but the effect is of similar magnitude in the PD and HC group. Conversely, an increase in the rate of saccades over time would be expected due to fatigue. This is observed to some extent and somewhat more pronounced in the PD compared to the HC group. Yet, the saccade rate in the PD group is consistently higher than in HC, from the first to the last trial.

In the PD group, MMSE scores correlated moderately with the median fixation duration (rho = 0.361; *p* = 0.01), longest fixation period (rho = 0.324; *p* = 0.022) and saccade rate (rho = –0.313; *p* = 0.028) as well as gaze parameters: SD of the horizontal gaze position (rho = –0.439; *p* = 0.001), MAD of the horizontal (rho = –0.482; *p* < 0.001) and vertical (rho = –0.473; *p* = 0.001) gaze position, QD of horizontal (rho = –0.457; *p* = 0.001) and vertical (rho = –0.441; *p* = 0.001) gaze position, and BCEA (rho = 0.316; *p* = 0.026). No correlation was found between the rest of the cognitive tests performed and eye-movement parameters.

Looking further for differences between male and female participants in the cohort, both mean and median pupil size difference was statistically significant (*p* = 0.01 and *p* = 0.008 respectively), with women presenting with larger pupil size (mm) than men during the task (median (IQR): 2.51 (0.28) vs 2.35 (0.28) and 2.59 (0.28) vs 2.46 (0.29) respectively).

### Logistic regression analysis

**Fig. 3:**
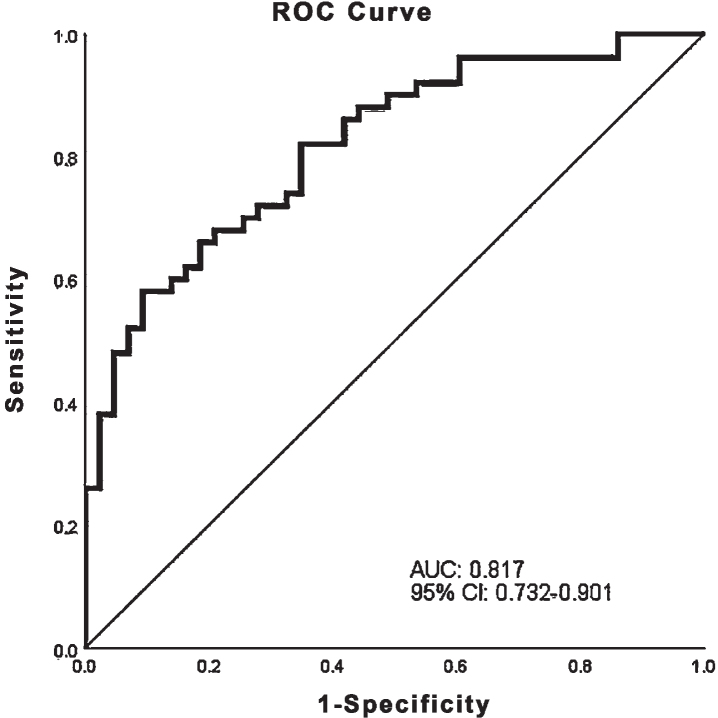
Receiver Operating Characteristic (ROC) curve of the multivariate model that includes longest fixation period, median pupil size, sex, and visuospatial/executive subscore of the Montreal Cognitive assessment test. AUC, Area under the curve; CI, Confidence interval.

Significant predictors of diagnosis in univariate models were: longest fixation period (OR 0.874; 95% CI 0.779–0.98; *p* = 0.021), mean and median pupil size (OR 0.798; 95% CI 0.664–0.939; *p* = 0.007 and OR 0.794; 95% CI 0.669–0.943; *p* = 0.008 respectively), saccade rate (OR 1.476; 95% CI 1.042 = 2.09; *p* = 0.028), visuospatial/executive score (OR 0.514; 95% CI 0.32–0.825; *p* = 0.006), sex (OR 3.276; 95% CI 1.398–7.674; *p* = 0.006). The final model included the median pupil size (OR 0.811; 95% CI 0.666–0.987; *p* = 0.037), longest fixation period (OR 0.798; 95% CI 0.691–0.921; *p* = 0.002), sex (OR 4.35; 95% CI 1.516–12.483; *p* = 0.006), visuospatial/executive score in MoCA (OR 0.422; 95% CI 0.233–0.764; *p* = 0.004). In all univariate and multivariate models we transformed the mean and median pupil size by multiplying by ten; the models, therefore, refer to mean and median pupil size in mm/10. ROC analysis was used to visualize the multivariate model’s potential to separate between HC and PD. The area under the ROC was determined to 0.817; 95% CI 0.732–0.901 (Fig. 3).

To assess residual confounders, conditional multivariate logistic regression analysis was repeated in a subgroup of 28 PD patients and 28 HC matched for age (+/- 1 year) and sex, and the results remained unchanged with OR for median pupil size 0.57, 95% CI 0.33–0.98 (*p* = 0.04), and OR for longest fixation period 0.67, 95% CI 0.46–0.99 (*p* = 0.04).

## DISCUSSION

In our study, we report the results of eye-movement measurements, and more specifically parameters of the fixation task examined with eye-tracker, as potential diagnostic biomarkers in early-stage PD. We found that HC kept their eyes more stable during a longer period on a stable target, whereas fixation was interrupted more easily in PD patients. Additionally, during effort and in stable luminance conditions, PD patients’ pupils were smaller than those of HC.

The fixation task required participants to keep their gaze on a dark target in the middle of a white screen for approximately three minutes, with short pauses when the target disappeared. Since the contrast between the background and the target, a simple dot, was sharp, and given that vision was corrected to normal, identifying the target, itself, was not challenging. Our results showed that PD patients were easily distracted during fixation; a higher median saccade rate and a shorter median longest fixation duration point to the same conclusion. This is in accordance with previous studies that have also reported higher distractibility and frequent saccadic intrusions during fixation in PD patients compared to HC, independently of cognitive status and PD stage [2, 12, 13]. Overall, PD patients disengage their attention easily compared to healthy adults. This phenomenon has widely been studied in order to investigate its relationship with anatomical and functional structures. Inhibition of unnecessary eye movements requires the involvement of cortical (dorsolateral prefrontal cortex) and subcortical (superior colliculus) brain structures [14] as well as an intact connection between the basal ganglia and these structures. These connective loops have been described to be impaired in PD, thus generating abnormal saccades, some of which are SWJ that are commonly seen in PD, even in early phases of the disease [2, 5]. Moreover, it has previously been suggested that increased inhibition upon the SC leads to a compensatory increase of the frontal eye field activity resulting in SWJ in PD [5]. In another study [15], it was shown that the frequency of saccadic intrusions during the oculomotor examination was negatively correlated with brain volume in PD patients. Finally, in a study on PD patients that had undergone unilateral pallidotomy [16], increased number and amplitude of SWJ was reported during sustained fixation, possibly attributed to alterations in the function of frontal and prefrontal cortical areas following the disruption of pallidal influence on the thalamocortical loops. Such studies on fixation analysis highlight the importance of investigating ocular fixation in PD.

Although the participants needed to maintain attention during the task, fixation per se is not cognitively demanding to the same extent as other common eye movement tasks (e.g., antisaccades), thus, large fati-gue effect was not expected. Our analysis has confirmed this assumption. If fatigue was driving the results, we would expect to see an increasing or decreasing trend over the trials in different parameters, but this was not observed.

Although an increase in saccadic intrusions and a decrease in fixation periods with age has been described in healthy populations with an age range between 21 and 81 years [17], we were not able to find such a correlation neither in the HC nor in the PD group. This might be attributed to the relatively narrower age range in our sample (42–77 years in the HC, 42–76 years in the PD group).

Regarding cognition, fixation and gaze parameters correlated weakly with MMSE but not with MoCA score in the PD group only, which may be attributed to the fact that all participants had good cognitive performance (i.e., high scores in both tests) combined with the ceiling effect of MMSE score [18]. MoCA has widely been recognized as a superior screening tool in PD as it includes executive function testing, while MMSE lacks this part [18].

Another interesting finding in our analysis was that both mean and median pupil size were significantly larger in the HC group than in the PD group. This poses significant questions on the role of the autonomous nervous system on the modulation of pupil size during cognitively demanding tasks in health and disease. The dilator and sphincter muscles of the pupil are controlled by the sympathetic and parasympathetic branches of the nervous system via neurotransmission that is done with catecholamines and acetylcholine, respectively. Sympathetic fibers innervate the dilator muscle evoking mydriasis, whereas inhibition of parasympathetic activity has the same effect by reducing constriction of the sphincter muscle [19]. However, the difference between the parasympathetic and sympathetic pathways is remarkable. While the parasympathetic pathway is mainly responsible for the luminance-based pupil constriction, and the circuit is short, in contrast, the sympathetic pathway driving mydriasis involves multiple cortical and subcortical domains such as the frontal cortex, the hypothalamus, the locus coeruleus as well as the spinal cord (C8-T2) [19]. Modulation of the norepinephrine and acetylcholine systems is done by cortical and subcortical areas that are involved in cognitive control and attention. Here lies the role of the locus-coeruleus norepinephrine (LC-NE) system that modulates mydriasis with respect to task demands [19–21] and affects the pupil both directly, increasing the sympathetic activity, and indirectly by sending inhibitory projections to the parasympathetic Edinger-Westphal nucleus that normally causes miosis. The complexity of the aforementioned pathway, as well as the functional role of the LC-NE in cognitive processes [21] explains how pupil dilation is related not only with luminance but also to cognitive factors such as attention, memory and cognitive load, prediction, decision, as well as autonomic activity. During the fixation paradigm, in stable light conditions, trying to focus on the target for almost three minutes with only short intervals of rest was demanding on an attentional level. Based on our knowledge of the physiological mechanisms that underlie pupil size regulation, pathology of the LC-NE in PD [22] is one possible mechanism that explains why PD patients’ pupils were smaller than HC’s during sustained fixation. However, apart from the role of pre-ganglionic structures of the autonomous nervous system, speculations can also be made on the role of the post-ganglionic innervation of the pupil based on previous descriptions of the post-ganglionic deficiency of the sympathetic and parasympathetic system that affect pupil size modulation in PD [23].

It is important to consider the possible effect of concomitant medication and comorbidities that may have played an important role in our findings. Depression was, not surprisingly, more common in the PD group, although the difference did not reach statistical significance (*p* = 0.07). Treatment with antidepressants is expected to cause mydriasis as a side effect; our PD group, however, presented with smaller pupil size. Additionally, the use of benzodiazepines and anticholinergic medication that could have an effect on the pupil size was more common in the PD group, yet, the differences lacked statistical significance. Diabetes may affect the pupil size but was only present in one HC. Finally, regarding the differences in urinary incontinence and/or prostate hyperplasia, the finding, itself, can be explained by the fact that urinary incontinence can be part of the non-motor symptoms in PD and by the anequal sex distribution in the two groups. Six PD patients were treated with medication that could possibly affect pupil size (solifenacine, alfuzosin, doxazosin, finasteride) vs only one in the HC group, a difference, however, the difference was not statistically significant.

Based on previous literature that used pupillometry to investigate sex differences during cognitive effort for similar behavioral performance [24, 25], we included some additional comparisons between male and female participants with respect to pupil size during the fixation task. Our results are in accordance with previous studies that show that women present with larger pupils while putting cognitive effort [24] while other studies show no difference between sexes [25]. Results should therefore be interpreted with caution and might be attributed to multiple factors.

### Limitations

Our study has several limitations. Sex distribution differed significantly between HC and PD group; however, sex was included in the multivariate model in our analysis in order to adjust for this parameter. Also, the clinical evaluation of PD patients was performed only in the ON-medication state, thus preventing the investigation of the effect of levodopa treatment on saccade rate and ocular fixation characteristics. However, as an indirect indicator, we didn’t find any significant correlations between the eye-movement parameters and the amount of levodopa equivalent intake. Finally, we were not able to precisely characterize the nature of the saccadic intrusions that interrupted fixation. Although we assume, based on the literature, that they were SWJ, they could as well be reflexive saccades or microsaccades. However, in our study we aimed to study sustained fixation in a natural and ecologically valid setting where the participant is free to move. Studies that have investigated such movements have used a very rigid eye tracker set-up with chin- and forehead rest and sometimes even a bite-bar. It is unclear whether SWJ and microsaccades can be reliably detected in a set-up without head-restraint due to the potential confounding with small head movements.

## CONCLUSION

Eye-tracking is a quick and easy method to investigate brain function, with only slight, if any, discomfort to the participant. In this case-control study, eye-tracking based measurements of gaze fixation and pupil reaction could be used to discriminate PD patients with short disease duration from healthy, age-matched participants. Our study suggests that PD diagnosis at an early stage can be aided by using oculomotor tests such as fixation and pupil reaction as physiological biomarkers, although larger studies are needed in order to evaluate the generalizability of the results.

## CONFLICT OF INTEREST

Benfatto, M. N. and Seimyr, G. Ö. own equity and receive salary from Optolexia, a company whose aim is to offer new technologies for the assessment of based on eye tracking and artificial intelligence. The venture is a result of projects funded by Sweden’s innovation agency – VINNOVA – (2014-03459; 2017-02317) and Karolinska Institutet Innovations.

PS receives funding from Vinnova, the Stockholm County Council, and Wallenberg Clinical Scholarship, as well as honoraria from Abbvie.

IM receives funding from Stockholm County Council (20180200), Neuro Fund Stockholm and Parkinson Research Foundation, Stockholm
